# Parallel Endografting And Chimney Endovascular (PEACE) registry outcomes in emergency repair of complex abdominal aortic aneurysms

**DOI:** 10.1093/bjs/znaf278

**Published:** 2025-12-30

**Authors:** Angelos Karelis, Donald Adam, Michele Piazza, Giovanni Tinelli, Enrico Gallito, Miltiadis Matsagkas, Barend Mees, Kevin Mani, Mario D’Oria, Wassim Mansour, Enrico Cieri, Tilo Kölbel, Björn Sonesson, Nuno V Dias, PEACE Contributors, Abdulrahman Husain, Francesco Squizzato, Simona Sica, Yamume Tshomba, Mauro Gargiulo, Konstantinos Tzimkas-Dakis, Stefanie Burger, Geert Willem Schurink, Anders Wanhainen, Peter Legeza, Andreina Monfreda, Sandro Lepidi, Marta Ascione, Luca di Marzo, Fino Gianluigi

**Affiliations:** Department of Clinical Sciences Malmö, Lund University, Malmö, Sweden; Vascular Centre, Department of Thoracic Surgery and Vascular Diseases, Skåne University Hospital, Malmö, Sweden; Department of Vascular Surgery, University Hospitals Birmingham NHS Foundation Trust, Birmingham, UK; Division of Vascular and Endovascular Surgery, Department of Cardiac, Thoracic, Vascular Sciences and Public Health, Padua University School of Medicine, Padua, Italy; Unit of Vascular Surgery, Fondazione Policlinico Universitario A. Gemelli IRCCS, Università Cattolica del Sacro Cuore, Rome, Italy; Vascular Surgery, University of Bologna, DIMEC, Bologna, Italy; Vascular Surgery Unit, IRCCS Sant’Orsola, Bologna, Italy; Department of Vascular Surgery, Larissa University Hospital, Faculty of Medicine, University of Thessaly, Larissa, Greece; Department of Vascular Surgery, Maastricht University Medical Centre, Maastricht, The Netherlands; Division of Vascular Surgery, Department of Surgical Sciences, Uppsala University, Uppsala, Sweden; Division of Vascular and Endovascular Surgery, Cardiovascular Department, University Hospital of Trieste, Trieste, Italy; Department of General Surgery and Surgical Specialties, Sapienza University of Rome, Rome, Italy; Vascular and Endovascular Surgery Unit University of Perugia, Ospedale S. Maria Della Misericordia, Perugia, Italy; German Aortic Centre Hamburg, Department of Vascular Medicine, University Heart and Vascular Center UKE Hamburg, University Medical Centre Hamburg-Eppendorf, Hamburg, Germany; Department of Clinical Sciences Malmö, Lund University, Malmö, Sweden; Vascular Centre, Department of Thoracic Surgery and Vascular Diseases, Skåne University Hospital, Malmö, Sweden; Department of Clinical Sciences Malmö, Lund University, Malmö, Sweden; Vascular Centre, Department of Thoracic Surgery and Vascular Diseases, Skåne University Hospital, Malmö, Sweden

## Abstract

**Background:**

Chimney endovascular aneurysm repair (chEVAR) techniques have been described to manage a group of patients who are unsuitable for either open aortic aneurysm surgery or a variety of standard endovascular repair techniques. The aim of this study was to assess the long-term clinical outcomes of chEVAR in emergency settings for patients with abdominal aortic aneurysms that have complex morphology.

**Methods:**

This was a multicentre retrospective study that included all consecutive patients undergoing urgent chEVAR with at least one chimney/parallel graft up to June 2021. Outcomes that were captured included 30-day mortality, long-term overall survival, aneurysm-related mortality, chimney-related complications, and target vessel patency.

**Results:**

Some 118 patients (mean(s.d.) age of 77(8) years; 72.0% male) underwent urgent or emergency chEVAR, 78 (66.1%) due to aortic rupture. The mean(s.d.) number of chimneys used per patient was 1.6(0.7). Technical success was achieved in 90.6% of patients, with a 30-day mortality rate of 17.7%. The mean follow-up was 4(3) years. Estimated overall survival was 69 ± 5% at 3 years, 45 ± 6% at 5 years, and 32 ± 6% at 7 years. Freedom from aneurysm-related mortality was 58 ± 6% at 5 years and 53 ± 6% at 7 years. In patients surviving the perioperative 30-day interval, freedom from aneurysm-related mortality was 73 ± 6% at 5 years and 66 ± 7% at 7 years. Primary target vessel patency at 5 and 7 years was 87 ± 4%, with renal arteries most frequently affected. Late reinterventions occurred in 16.1% of patients, mostly for type Ia endoleaks (8 of 25 reinterventions (32%)) and type Ib endoleaks (5 of 25 reinterventions (20%)).

**Conclusion:**

In this Parallel Endografting And Chimney Endovascular (PEACE) registry study, chEVAR was associated with a high rate of technical success and acceptable early outcomes, but, in the longer term, was associated with high rates of reintervention and mortality. It appears to represent a reasonable alternative technique for patients presenting as an emergency with complex aortic aneurysm morphology when standard open and endovascular techniques are not feasible.

## Teaser

This study from the Parallel Endografting And Chimney Endovascular (PEACE) registry presents the largest cohort to date examining chimney endovascular aneurysm repair (chEVAR) in the emergency repair of complex abdominal aortic aneurysms. It demonstrates high technical success and acceptable early outcomes, while also highlighting the long-term challenges of mortality and reintervention in this high-risk population.

## Introduction

The treatment of abdominal aortic aneurysms (AAA) has seen remarkable advancements in recent years, particularly with the advent of minimally invasive endovascular aneurysm repair (EVAR) techniques. These innovations have expanded therapeutic options for patients with severe comorbidities who are unfit for open surgical repair^1^. With the declining use of open surgical repairs for ruptured AAA due to high mortality rates^2–4^, the need for an off-the-shelf endovascular solution has become increasingly apparent—particularly in the most challenging cases of ruptured or emergency complex AAA. This unmet need led to the development of chimney endovascular aneurysm repair (chEVAR), which was initially introduced as a bailout technique for intraoperative rescue of occluded renal arteries and later adopted for the treatment of complex AAA^5^.

Initially introduced as a bailout option in acute settings, chEVAR has since gained popularity as a versatile and readily available solution for managing complex aortic aneurysms, particularly when custom-made devices are unavailable, offering the advantage of requiring no graft modifications. However, long-term complications—most notably proximal type Ia endoleaks caused by gutter seal failure—remain a critical concern, particularly in ruptured or symptomatic AAA. While short-term and mid-term outcomes of chEVAR procedures have been documented^6^, robust data on long-term outcomes remain limited^7^, especially for acute or emergency procedures^8^. This underscores the ongoing need for additional evidence focusing specifically on acute and emergent presentations.

The Parallel Endografting And Chimney Endovascular (PEACE) registry was established to address these limitations by comprehensively evaluating long-term clinical outcomes, including mortality and morbidity, in patients undergoing emergent chEVAR procedures. It also investigates chimney and parallel graft patency, the incidence of endoleaks, and the frequency of reinterventions during extended follow-up. By filling these gaps, the PEACE registry aims to provide a deeper understanding of the durability, effectiveness, and safety of chEVAR techniques in managing complex aortic pathologies in acute and emergency settings.

## Methods

### Study design

The PEACE registry is a multicentric retrospective registry involving high-volume centres (>30 aortic cases per year) specializing in complex endovascular aortic repair. Data were collected exclusively from hospital medical records and anonymized before being entered into a standardized data collection sheet by each participating centre.

Consecutive patients who underwent EVAR using at least one chimney or parallel endograft technique for complex AAA from April 2010 until June 2021 were included in the study. Eligible patients were adults presenting with acute aortic pathologies involving complex AAA, defined as aneurysms involving the visceral arteries or infrarenal with a short or severely angulated proximal neck <10 mm. Inclusion required treatment with chEVAR and availability of long-term follow-up data. Patients undergoing standard EVAR without the use of chimney or parallel techniques or those with inadequate follow-up data were excluded from the study.

The study was conducted in accordance with the ethical principles outlined in the Declaration of Helsinki and complied with the STROBE guidelines for cohort studies^9^. The study protocol was approved by the institutional ethical committees locally or ethical approval was waived in accordance with the local ethical committee. All participating centres performed procedures in accordance with their respective institutional and national regulatory frameworks governing the use of off-label endovascular devices, including the consent procedure. Each centre retained responsibility for obtaining the necessary local approvals and for ensuring compliance with ethical and governance standards.

### Procedures and follow-up protocols

Procedures were performed at physicians’ discretion and according to the institutional standard of care with operative techniques tailored to individual patient anatomy, clinical presentation, and device availability. This included the anaesthesia type, vascular access, devices used, and procedural techniques.

Follow-up was performed according to each centre’s routine, including the decision to reintervene. The study protocol included information from a computed tomography angiography (CTA) before discharge or within 1 month of EVAR and yearly thereafter.

### Outcomes and definitions

Definitions were standardized according to established reporting standards for endovascular aortic repair^10^. The primary endpoints of the study were 30-day mortality, long-term overall survival, freedom from aneurysm-related mortality, adverse events related to chimneys or parallel grafts, and primary target vessel patency, and the secondary endpoints of the study were reintervention rates, the incidence of all types of endoleaks (including types Ia, Ib, and III), target vessel-related complications, aneurysm sac regression or stability, and clinical success. Target vessel-related complications were defined as any target vessel-related complication leading to aneurysm rupture, death, occlusion, component separation, or reintervention to maintain target vessel patency or to treat a target vessel-related component separation or endoleak^11^. Arteries that were intentionally covered were not considered as treatment failure.

Sac regression was defined as a decrease in sac diameter of ≥5 mm, stability as no significant change, and growth as an increase of >5 mm. Outcomes were classified as early if they occurred within 30 days of the procedure and late if they occurred afterwards. Overall mortality encompassed both early and late deaths.

### Statistical analysis

Categorical data are presented as *n* (%) and continuous data are presented as mean(s.d.). The *t* test was used for comparisons of continuous data. Time-dependent outcomes were analysed using Kaplan–Meier estimates with standard errors. Missing data were addressed using valid percentages for variables with <10% missing values. Cases or variables with >10% missing data were excluded from the analysis to maintain data integrity and minimize potential bias. Univariable regression analyses were conducted to identify factors associated with 30-day mortality and type Ia endoleak at completion angiography. *P* < 0.050 (two tailed) was considered statistically significant. The survfit function in the R package survival was used to perform the competing-risk analyses (R, R Core Team, R Foundation for Statistical Computing, Vienna, Austria, 2025). All other statistical analyses were conducted using SPSS^®^ (IBM, Armonk, NY, USA; version 30).

## Results

### Baseline characteristics

Some 118 patients undergoing acute or emergency chEVAR between 2010 and 2021 were included from 12 aortic centres (*Table S1*). The mean(s.d.) age was 77(8) years and 85 of the patients (72.0%) were male. Detailed baseline characteristics are provided in *Table 1*.

**Table 1:** Demographics and clinical characteristics for 118 patients treated with chEVAR in an acute setting of complex AAA

Characteristic	Total (*n* = 118)	Ruptured (*n* = 78)	Symtomatic (*n* = 40)	*P*
Age (years), mean(s.d.)	77(8)	75(8)	76(9)	0.50
Male	85 (72.0)	57 (73)	28 (70)	0.72
BMI (kg/m^2^), mean(s.d.)	26(4)	26(4)	25(5)	0.53
**Smoking**	56 (47.4)	33 (42)	22 (55)	0.01*
Prior	39 (33.0)	23 (29)	16 (40)	0.04*
Current	17 (14.4)	11 (14)	6 (15)	0.78
Hypertension	86 (72.8)	58 (75)	28 (72)	0.68
Hyperlipidaemia	49 (41.5)	32 (42)	17 (44)	0.83
**Coronary artery disease**	48 (40.6)	28 (36)	18 (45)	0.31
PCI	15 (12.7)	9 (11)	6 (15)	0.59
CABG	20 (16.9)	12 (16)	8 (21)	0.51
Congestive heart failure	18 (15.2)	10 (14)	8 (21)	0.38
COPD	27 (22.9)	15 (20)	12 (32)	0.16
Peripheral artery disease	17 (14.4)	9 (12)	8 (21)	0.23
Diabetes mellitus	19 (16.1)	9 (12)	10 (26)	0.05
Stroke	9 (7.6)	8 (11)	1 (3)	0.13
TIA	5 (4.2)	4 (6)	1 (3)	0.47
**Chronic kidney disease**				
Stage III–V	56 (47.4)	33 (43)	23 (58)	0.15
Creatinine (µmol/l), mean(s.d.)	115(69)	117(80)	109(39)	0.31
eGFR (ml/min/1.73 m^2^), mean(s.d.)	59(26)	62(30)	54(18)	0.04*
**ASA grade**				
I–III	46 (38.9)	26 (48)	20 (67)	0.04*
IV–V	38 (32.2)	28 (52)	10 (33)	0.07

Values are *n* (%) unless otherwise indicated. *Statistically significant. chEVAR, chimney endovascular aneurysm repair; AAA, abdominal aortic aneurysms; PCI, percutaneous coronary intervention; CABG, coronary artery bypass graft surgery; COPD, chronic obstructive pulmonary disease; TIA, transient ischaemic attack; eGFR, estimated glomerular filtration rate.

Of the cohort, 21 patients (18%) had undergone prior EVAR and 5 patients (4%) had a history of prior open aortic repair. The mean(s.d.) maximum diameter of aortic aneurysms was 73(19) mm. Most aneurysms were contained ruptures (70 of 118 patients (59.3%)) or symptomatic but non-ruptured (40 of 118 patients (33.8%)), while free ruptures occurred in 8 of 118 patients (6.7%). Juxtarenal aneurysms were most common (56 of 118 patients (47.4%)), followed by infrarenal aneurysms (31 of 118 patients (26.2%)) and pararenal aneurysms (31 of 118 patients (26.2%)).

Regarding internal iliac artery status, of 118 patients, both arteries were patent in 93 patients (78.8%), one artery was occluded in 14 patients (11.8%), and both arteries were occluded in 4 patients (3.3%). Among renovisceral vessels, 97 of 118 patients (82.2%) had all vessels patent, while 21 of 118 patients (17.7%) had at least one vessel occluded. Anatomic characteristics are detailed in *Table 2*.

**Table 2:** Anatomic characteristics and indications for repair for 118 patients treated with chEVAR in an acute setting of complex AAA

Characteristic	Total (*n* = 118)	Ruptured (*n* = 78)	Symtomatic (*n* = 40)	*P*
**Prior aortic repair**				
OAR	5 (4.2)	2 (3)	3 (8)	0.24
EVAR	21 (17.7)	8 (10)	13 (33)	0.03*
F/BEVAR	1 (0.8)	1 (1)	0 (0)	0.47
Largest diameter of aortic aneurysm (mm), mean(s.d.)	73(19)	73(20)	72(17)	0.59
**Status of aneurysm**				
Symptomatic non-ruptured	40 (33.8)	0 (0)	40 (100)	<0.01*
Contained ruptured	70 (59.3)	70 (90)	0 (0)	<0.01*
Free rupture	8 (6.7)	8 (10)	0 (0)	<0.01*
**Type of aneurysm**				
Infrarenal	31 (26.2)	21 (27)	10 (25)	0.84
Juxtarenal	56 (47.4)	36 (46)	20 (50)	0.71
Pararenal	31 (26.2)	21 (27)	10 (25)	0.87
**Status of hypogastric arteries**				
Both patent	93 (78.8)	62 (86)	31 (79)	0.46
One occluded	14 (11.8)	7 (10)	7 (18)	0.13
Both occluded	4 (3.3)	3 (4)	1 (3)	0.93
**Status of renovisceral target vessel**				
All patent	97 (82.2)	55 (81)	32 (80)	0.96
At least one occluded	21 (17.7)	13 (19)	8 (20)	0.58
**Number of target vessels, mean(s.d.)**	1.6(0.7)	1.6(0.7)	1.7(0.7)	0.46
1	62 (52.5)	44 (56)	18 (45)	0.07
2	42 (35.5)	25 (32)	17 (43)	0.64
3	9 (7.6)	6 (8)	3 (8)	0.73
4	2 (1.6)	2 (3)	0 (0)	0.54

Values are *n* (%) unless otherwise indicated. *Statistically significant. chEVAR, chimney endovascular aneurysm repair; AAA, abdominal aortic aneurysms; OAR, open aortic repair; EVAR, endovascular aortic repair; F/BEVAR, fenestrated/branched EVAR.

### Procedural data

**Fig. 1:**
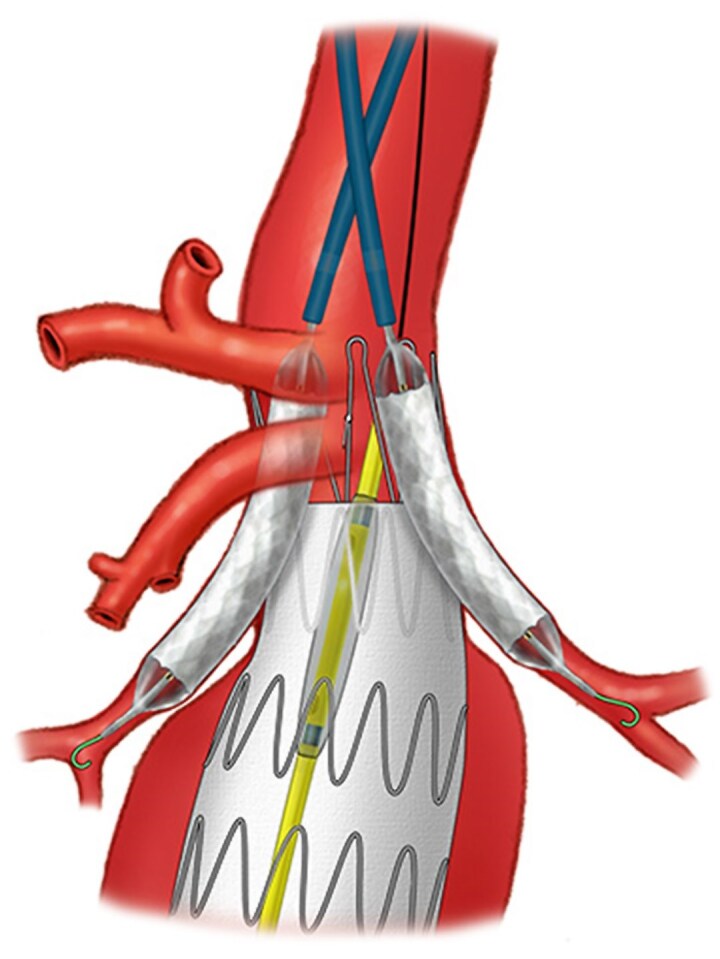
Illustration of a chEVAR with two renal chimneys performed during emergency repair of a juxtarenal aortic aneurysm chEVAR, chimney endovascular aneurysm repair.

A total of 181 chimneys were performed, a mean(s.d.) of 1.6(0.7) per patient (*Table 2*, *Table 3*, and *Fig. 1*). Of 118 patients, one target vessel was treated in 62 patients (52.5%), two target vessels were treated in 42 patients (35.5%), three target vessels were treated in 9 patients (7.6%), and four target vessels were treated in 2 patients (1.6%). Renal arteries were the most frequent targets (76.2%; 138 chimneys), the right renal artery (RRA) was sometimes the target (19.9% (36 chimneys)), and the left renal artery (LRA) was sometimes the target (39.8% (72 chimneys)).

**Table 3:** Procedural plan and device design for 118 patients treated with chEVAR in an acute setting of complex AAA

Characteristic	Total (*n* = 118)	Ruptured (*n* = 78)	Symtomatic (*n* = 40)	*P*
**Proximal sealing zone**				
Zone 3	2 (1.7)	2 (3)	0 (0)	0.54
Zone 4	8 (6.8)	4 (5)	4 (10)	0.21
Zone 5	4 (3.4)	2 (3)	2 (5)	0.96
Zone 6	20 (17.2)	17 (22)	3 (8)	0.01*
Zone 7	43 (37.0)	33 (42)	10 (25)	0.04*
Zone 8	36 (31.0)	17 (22)	19 (48)	<0.01*
Zone 9	3 (2.5)	1 (1)	2 (5)	0.98
**Distal sealing zone**				
Zone 9—infrarenal	21 (18.5)	12 (16)	9 (24)	0.72
Zone 10—CIA level	81 (71.6)	54 (72)	27 (71)	0.79
Zone 11—EIA level	11 (9.7)	9 (12)	2 (5)	0.25
**Intentional coverage of visceral vessel**	35 (29.6)	27 (35)	8 (20)	0.10
RRA/accessory RRA	14 (11.8)/1 (0.8)	14 (18)	1 (3)	0.02*
LRA/accessory LRA	20 (16.9)/1 (0.8)	16 (21)	5 (13)	0.19
Coeliac trunk	4 (3.3)	2 (3)	2 (5)	0.96
SMA	1 (0.8)	0 (0)	1 (3)	0.86
Total vessels, *n*	41			
**Graft design**				
EVAR	98 (83.0)	67 (86)	31 (78)	0.41
TEVAR	17 (14.4)	11 (14)	6 (15)	0.74
Aortic cuff	3 (2.5)	0	3 (8)	0.09
**Aortic stent graft**				
Cook Flex/Alpha	44 (37.2)	34 (44)	10 (25)	0.04*
Medtronic Endurant	35 (29.6)	19 (24)	16 (40)	0.06
Gore Excluder	17 (14.4)	10 (13)	7 (18)	0.55
Cook—TEVAR	15 (12.7)	9 (12)	6 (15)	0.73
Gore Conformable	3 (2.5)	3 (4)	0	0.29
Other—TEVAR (Lombard, Valiant, Gore TAG)	3 (2.5)	3 (4)	0	0.29
Main device delivery system (Fr), mean(s.d.)	19(4)	19(4)	19(3)	0.85
Device proximal diameter (mm), mean(s.d.)	32(4)	32(4)	32(4)	0.46
Main aortic device proximal oversizing (%), mean(s.d.)	23(8)	24(7)	22(8)	0.36

Values are *n* (%) unless otherwise indicated. *Statistically significant. chEVAR, chimney endovascular aneurysm repair; AAA, abdominal aortic aneurysms; CIA, common iliac artery; EIA, externa iliac artery; RRA, right renal artery; LRA, left renal artery; SMA, superior mesenteric artery; EVAR, endovascular aneurysm repair; TEVAR, thoracic endovascular aneurysm repair.

Intentional coverage of at least one renovisceral vessel was performed in 35 of 118 patients (29.6%), with most of the vessels being renal or accessory renal arteries. Of 181 chimneys, balloon-expandable covered stents were used in 141 chimneys (77.9%), self-expandable covered stents were used in 37 chimneys (20.4%), and balloon-expandable non-covered stents were used in 3 chimneys (1.6%) (*Table S2*).

The mean(s.d.) estimated blood loss was 400(931) ml. Aortic occlusion balloons were used in 27 of 118 patients (22.8%). One case required an adjunctive procedure with laparotomy due to abdominal compartment syndrome, with abdominal VAC treatment initiated. Three intraoperative deaths (2.5%) were reported; all due to hypovolaemic shock. The mean(s.d.) hospital stay was 9(13) days and the mean(s.d.) ICU stay was 2(6) days, both longer in patients presenting with ruptured aneurysms (*P* < 0.001 for both).

### Early outcomes

Operative technical success was achieved in 107 of 118 patients (90.6%) (*Table 4*). Completion angiography revealed type Ia endoleaks in 11 patients (9.3%) and type III endoleaks in 1 patient (0.8%). No statistically significant association was observed between the number of chimneys and the occurrence of type Ia endoleak at completion angiography when analysed as an independent risk factor (*Table S3*).

**Table 4:** Procedural details for 118 patients treated with chEVAR in an acute setting of complex AAA

Characteristic	Total (*n* = 118)	Ruptured (*n* = 78)	Symtomatic (*n* = 40)	*P*
**Anaesthesia**				
Local	21 (18.5)	16 (21)	5 (13)	0.16
Local converted into general	16 (14.2)	15 (19)	1 (3)	0.02*
General	75 (66.9)	45 (58)	30 (75)	0.19
**Upper extremity access**				
Open right/left/both	16 (13.5)/28 (23.7)/15 (12.7)	9 (12)/13 (17)/10 (13)	7 (18)/15 (38)/5 (13)	0.03*
Percutaneous right/left/both	31 (26.2)/12 (10.1)/5 (4.2)	21 (27)/8 (10)/4 (5)	10 (25)/4 (10)/1 (3)	0.90
**Percutaneous femoral access**				
Unilateral	11 (9.3)	6 (8)	5 (13)	0.23
Bilateral	79 (66.9)	52 (67)	27 (68)	0.87
Palmaz stent, yes	5 (4.2)	3 (4)	2 (5)	0.77
Total procedure time (min), mean(s.d.)	219(110)	243(111)	219(110)	0.80
Contrast volume (ml), mean(s.d.)	156(103)	188(88)	165(129)	0.04*
Fluoroscopy time (min), mean(s.d.)	57(44)	64(37)	60(58)	0.34
Dose area product (Gy/cm^2^), mean(s.d.)	420(366)	494(125)	351(519)	<0.01*
Completion cone beam CT, yes	23 (19.4)	17 (22)	6 (15)	0.39
Use of occlusion balloon, yes	27 (22.8)	22 (19)	4 (10)	0.04*
Estimated blood loss (ml), mean(s.d.)	400(931)	880(1081)	335(348)	0.12
Technical success	107 (90.6)	68 (87)	39 (98)	0.07

Values are *n* (%) unless otherwise indicated. *Statistically significant. chEVAR, chimney endovascular aneurysm repair; AAA, abdominal aortic aneurysms.

No target vessel or chimney occlusions were noted within 30 days. Early complications occurred in 49.1% of patients (58 of 118 patients) including a 30-day all-cause mortality rate of 17.7% (21 of 118 patients) (*Table 5*). Independent predictors of 30-day mortality were not identified on univariable analysis (*Table S3*); however, urgent repairs for ruptured aneurysms were significantly associated with mortality (*P* = 0.009).

**Table 5:** Postoperative and early outcomes for 118 patients treated with chEVAR in an acute setting of complex AAA

Characteristic	Total (*n* = 118)	Ruptured (*n* = 78)	Symtomatic (*n* = 40)	*P*
Intensive care duration (days), mean(s.d.)	2(6)	4(7)	1(2)	<0.01*
Hospitalization duration (days), mean(s.d.)	9(13)	13(15)	8(6)	<0.01*
All-cause 30-day death	21 (17.7)	19 (24)	2 (5)	<0.01*
Any complication within 30 days	58 (49.1)	42 (54)	15 (38)	0.17
Access site complication	17 (14.4)	9 (12)	8 (20)	0.16
New-onset CHF	11 (9.3)	9 (12)	2 (5)	0.26
Bowel ischaemia	3 (2.5)	3 (4)	0	0.45
Stroke	4 (3.3)	4 (5)	0	0.51
**Respiratory complication**	12 (10.1)	10 (12)	1 (1)	0.14
Respiratory failure without tracheotomy	1 (0.8)	1 (1)	0	0.47
Respiratory failure with tracheotomy	8 (6.7)	8 (10)	0	0.04*
Multiple (pneumonia/respiratory failure/prolonged intubation)	2 (1.6)	1 (1)	1 (1)	0.12
Myocardial infarction	3 (3.5)	1 (1)	2 (5)	<0.01*
**Renal impairment**	29 (24.5)	24 (30)	5 (13)	0.10
Severe acute renal function deterioration—no dialysis	15 (12.7)	11 (14)	4 (10)	0.32
Severe acute renal function deterioration—temporary dialysis	13 (11.0)	12 (15)	1 (3)	0.06
Permanent dialysis	1 (0.8)	1 (1)	0	0.47
Creatinine at discharge (μmol/l), mean(s.d.)	166(138)	172(147)	149(115)	0.32
**Spinal cord injury**	2 (1.6)	2 (2)	0	0.60
Sensory deficit	1 (0.8)	1 (1)	0	0.47
Motor deficit—non-ambulate	1 (0.8)	1 (1)	0	0.47
**Early reintervention**	23 (19.4)	16 (21)	7 (18)	0.80
Aortic or chimney related	9 (9.3)	7 (9)	2 (5)	0.72
Non-aortic/chimney related	14 (11.8)	10 (13)	4 (10)	0.78

Values are *n* (%) unless otherwise indicated. *Statistically significant. chEVAR, chimney endovascular aneurysm repair; AAA, abdominal aortic aneurysms; CHF, cardiac heart failure.

The most common complications, excluding deaths, were severe renal impairment (29 of 118 patients (24.5%)), access site complications (17 of 118 patients (14.4%)), respiratory complications (12 of 118 patients (10.1%)), and new-onset cardiac heart failure (11 of 118 patients (9.3%)). Three patients (2.5%) required bowel resection due to bowel ischaemia, one of whom had a chimney in the superior mesenteric artery, while three other patients (2.5%) experienced postoperative myocardial infarctions. Two patients (1.6%) had spinal cord injuries, one with partial recovery and one with persistent motor deficits.

Early reinterventions were needed in 23 of 118 patients (19.4%), with 9 patients (9.3%) requiring early interventions to correct aortic or chimney complications and 14 patients (11.8%) requiring early interventions for non-aortic/chimney-related issues (*Table S4*).

At discharge, of 104 patients, 47 (45.1%) received double antiplatelet therapy, 44 (42.3%) received single antiplatelet therapy (SAPT), 6 (5.7%) were prescribed warfarin, 5 (4.8%) received SAPT + low-dose direct oral anticoagulant (DOAC), and 2 (1.9%) received therapeutic-dose DOAC. Statins were prescribed to 86 of 104 patients (82.6%) and beta-blockers were prescribed to 45 of 104 patients (43.2%).

### Long-term outcomes

**Fig. 2:**
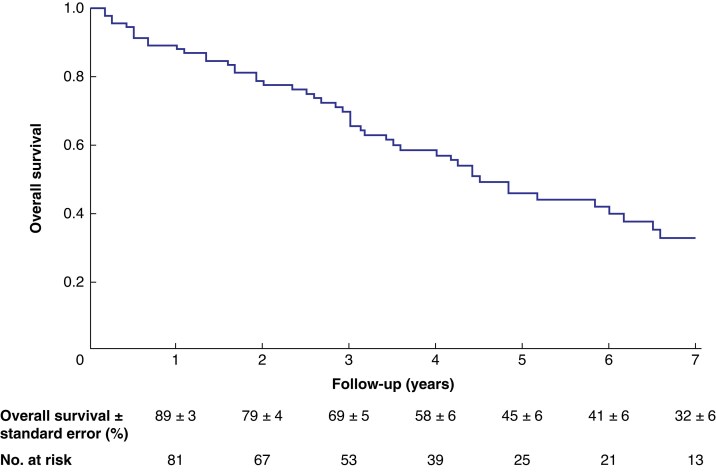
Estimated overall survival, excluding 30-day mortality, for patients who underwent urgent chEVAR for a complex AAA chEVAR, chimney endovascular aneurysm repair; AAA, abdominal aortic aneurysm.

**Fig. 3:**
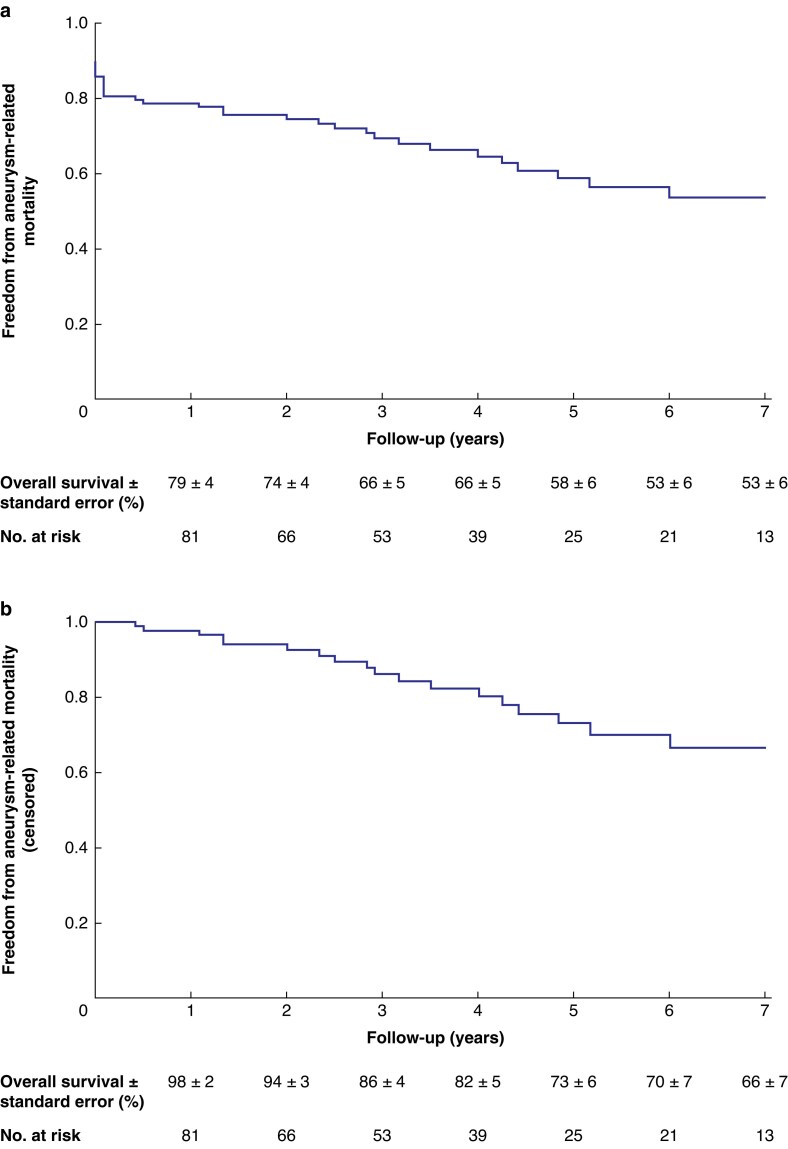
Estimated freedom from aneurysm-related mortality for patients who underwent urgent chEVAR for a complex AAA **a** Without censoring. **b** When 30-day mortality was censored. chEVAR, chimney endovascular aneurysm repair; AAA, abdominal aortic aneurysm.

The mean(s.d.) clinical follow-up duration was 4(3) years and the mean(s.d.) imaging follow-up duration was 3(2) years. Long-term outcome characteristics are summarized in *Table 6*. At the last follow-up, all-cause mortality was reported to be 63.5% (75 of 118 patients); 26 patients (22.0%) experienced aneurysm-related deaths, 40 patients (33.8%) experienced deaths that were unrelated to the aneurysm, and 9 patients (7.6%) experienced a death for which the cause was unknown. The estimated overall survival, excluding 30-day mortality, was 69 ± 5% at 3 years and 45 ± 6% at 5 years (*Fig. 2*). The estimated freedom from aneurysm-related mortality, without censoring, was 58 ± 6% at 5 years (*Fig. 3a*). When 30-day mortality was censored, freedom from aneurysm-related mortality improved to 73 ± 6% at 5 years (*Fig. 3b*).

**Table 6:** Long-term outcome characteristics for 118 patients treated with chEVAR in an acute setting of complex AAA

Characteristic	Total (*n* = 118)	Ruptured (*n* = 78)	Symtomatic (*n* = 40)	*P*
Death at last follow-up	75 (63.5)	52 (67)	23 (58)	0.33
Post-implantation aneurysm rupture	1 (0.8)	1 (1)	0	0.47
Open conversion	2 (1.6)	1 (1)	1 (3)	0.12
Chimney occlusion	8 (6.7)	6 (8)	2 (5)	0.02*
Graft infection	2 (1.6)	2 (3)	0	0.60
Myocardial infarction	1 (0.8)	1 (1)	0	0.47
Stroke	1 (0.8)	1 (1)	0	0.47
Acute limb ischaemia	1 (0.8)	1 (1)	0	0.47
New-onset CHF	1 (0.8)	1 (1)	0	0.47
Iatrogenic AV fistula in brachial artery	1 (0.8)	0	1 (1)	0.86
Creatinine at last follow-up (μmol/l), mean(s.d.)	172(147)	184(166)	154(118)	0.10
Creatinine increase *versus* preop (μmol/l), mean(s.d.)	57(78)	67(86)	45(79)	0.17
eGFR at last follow-up (ml/min/1.73 m^2^), mean(s.d.)	48(26)	46(27)	51(23)	0.27
eGFR decrease *versus* preop (ml/min/1.73 m^2^), mean(s.d.)	11(3)	16(35)	3(5)	<0.01*
Permanent new-onset dialysis	7 (5.9)	7 (9)	0	0.01*
At least one late reintervention	19 (16.1)	12 (10)	7 (18)	0.77
Total late reinterventions, *n*	25	17	8	0.83
**Aneurysm sac behaviour**				
Unchanged	36 (45.0)	23 (46)	13 (43)	0.68
Shrinkage (decrease in sac diameter of ≥5 mm)	36 (45.0)	22 (44)	14 (47)	0.83
Enlargement (increase in sac diameter of >5 mm)	8 (10.0)	5 (10)	3 (10)	0.27
Last follow-up sac diameter (mm), mean(s.d.)	64(21)	64(21)	64(19)	0.45

Values are *n* (%) unless otherwise indicated. *Statistically significant. chEVAR, chimney endovascular aneurysm repair; AAA, abdominal aortic aneurysms; CHF, cardiac heart failure; AV, arteriovenous.

**Fig. 4:**
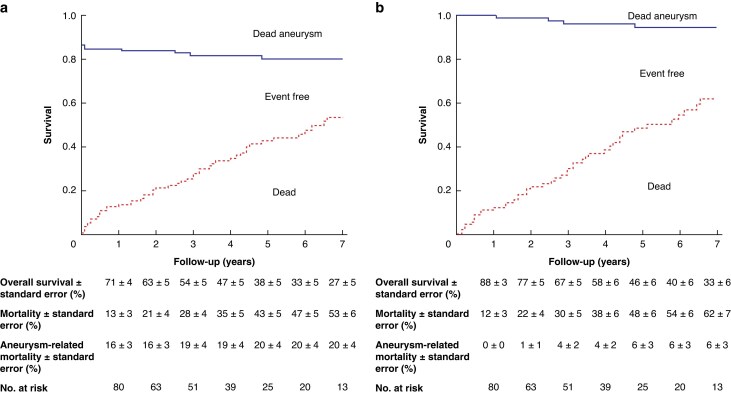
Competing-risk analysis of survival and aneurysm-related mortality after chEVAR **a** Including 30-day mortality, **b** Censoring 30-day mortality. Curves represent overall survival, aneurysm-related death, and non-aneurysm death. chEVAR, chimney endovascular aneurysm repair.

Competing-risk analysis demonstrated that when 30-day mortality was included, the estimated probability of aneurysm-related death was 19 ± 4% at 3 years and 20 ± 4% at 5 years, corresponding to a freedom from aneurysm-related mortality of approximately 80% at 5 years (*Fig. 4a*). When 30-day mortality was censored, the cumulative incidence of aneurysm-related death decreased to 4 ± 2% at 3 years and 6 ± 3% at 5 years, corresponding to a freedom from aneurysm-related mortality of approximately 94% at 5 years (*Fig. 4b*). Over the same follow-up, all-cause mortality increased to 43 ± 5% at 5 years (including early deaths) and 48 ± 6% at 5 years (when early mortality was censored).

Among the 26 aneurysm-related deaths reported during follow-up, the majority occurred within the first 30 days (21 of 118 patients (17.7%)); later aneurysm-related deaths (5 of 118 patients (4.2%)) included three instances (2.5%) of acute renal failure with concomitant respiratory failure, one instance (0.8%) of aortic rupture 30 months after surgery, and one instance (0.8%) of aortic graft infection 13 months after surgery.

Late type I endoleaks were observed in 9 of 118 patients (7.6%) during the follow-up; persistent type Ia endoleaks were identified in 7 patients (5.9%) and type Ib endoleaks were found in 2 patients (1.6%). All cases underwent reinterventions. Additionally, type II endoleaks with associated sac growth were observed in two patients (1.6%), persisting despite multiple embolization attempts. No other late endoleaks were detected. The estimated freedom from type Ia endoleaks at 5 years was 91 ± 4% (*Fig. S1*).

Late chimney occlusions occurred in 8 of 118 patients (6.7%), corresponding to a late chimney occlusion rate of 4.4% (8 of 181 chimneys). All occlusions affected renal chimneys, with the LRA being most frequently involved (5 patients). The mean(s.d.) time to occlusion was 20(13) months. Of the occlusions, three patients underwent recanalization attempts, all of which were unsuccessful. The estimated target vessel patency at 5 years was 87 ± 4% (*Fig. S2*).

Among all late complications, deterioration of renal function was the most common, with at least seven patients requiring permanent dialysis after surgery and one patient undergoing a kidney transplant. Of those, six had a renal chimney (5 involving LRA and 2 RRA, including one case with two renal chimneys), and one case involved intentional coverage of both renal arteries.

A total of 25 late reinterventions were performed in 19 of 118 patients (16.1%). Most of these reinterventions were performed to address type Ia endoleaks (8 of 25 reinterventions (32%)) and type Ib endoleaks (5 of 25 reinterventions (20%)) (*Table S5*).

Aneurysm sac behaviour at the last follow-up revealed that 36 of 80 patients (45%) had a sac that was unchanged in terms of size, 36 of 80 patients (45%) had a sac that demonstrated shrinkage (decrease in sac diameter of ≥5 mm), and 8 of 80 patients (10%) had a sac that exhibited enlargement (increase in sac diameter of >5 mm). The mean(s.d.) sac diameter at the last follow-up was 64(21) mm, representing a mean(s.d.) reduction of 9(3) mm or 12% (*P* < 0.001).

Finally, primary clinical success was estimated at 35 ± 5%, while secondary clinical success was 41 ± 5% at 5 years (*Fig. S3*).

## Discussion

The PEACE registry provides valuable insights into the long-term outcomes of emergency and urgent chEVAR for complex AAA. It is a feasible option for high-risk patients with complex aortic pathologies in emergency settings. While the procedure demonstrates high technical success and can achieve immediate aneurysm stabilization, the long-term results underscore the high risk of later failure, the need for rigorous postoperative monitoring of survivors due to high rates of reinterventions, and, potentially, the need for further advancements regarding technique and technology to improve outcomes.

The technical success rate of 90.6% is consistent with findings from other studies that included elective cases^6,7^, reinforcing the feasibility and effectiveness of chEVAR in managing complex aneurysms under urgent circumstances. One of the key advantages of chEVAR lies in its adaptability to challenging anatomies, such as short or inadequate proximal landing zones. While alternative techniques like physician-modified endografts (PMEG)^12^ or *in-situ* laser fenestrations^13^ have emerged, they require significantly longer to achieve exclusion of the aneurysm—an important limitation in urgent and emergency settings where rapid intervention is critical. In addition, the *in-situ* laser technique may be constrained by limited availability of laser equipment. These factors underscore the continued relevance of chEVAR in the acute management of complex aortic aneurysms.

The relatively high 30-day mortality rate (17.7%) reflects the high-risk nature of the patient cohort, many of whom presented with contained or free ruptures, and should be considered in the context of these emergency circumstances. This rate is comparable to other studies on emergency EVAR for non-complex ruptured aneurysms^14,15^ and appears to compare favourably to the reported outcomes for open repair in similar cases^16^.

The early complication rate (49.1%) and the need for early reinterventions (19.4%) are also indicative of the inherent risks of emergency aortic repair and underscore the complexity of these procedures and the vulnerability of the patient population. Severe renal impairment complications (24.5%) and access site complications (14.4%) were the most common early complications, emphasizing the physiological toll of the procedure and the importance of perioperative management strategies to mitigate these risks. The high incidence of severe renal impairment (24.5%) is particularly concerning and warrants further investigation into strategies for renal protection during and after chEVAR.

Long-term outcomes reveal several important findings. A freedom from aneurysm-related mortality of 73% at 5 years (when censoring 30-day mortality) suggests that chEVAR can offer protection against aneurysm-related death in survivors of the perioperative interval. However, this rate is lower than those reported in elective settings for similar complex anatomies treated with fenestrated EVAR^17^. This raises the question of whether chEVAR should be reserved for urgent cases where other options are not feasible. The overall survival rates, censored for 30-day mortality, were 69% at 3 years and 45% at 5 years, underscoring the substantial late mortality in this high-risk population.

The competing-risk analysis further refines the interpretation of these outcomes compared with traditional Kaplan–Meier methods. In the cause-specific Kaplan–Meier approach, non-aneurysm deaths are treated as censored, potentially underestimating freedom from aneurysm-related mortality. In contrast, the Aalen–Johansen estimator accounts for competing deaths, providing a more accurate crude probability of aneurysm-related death. The present results show a 5-year cumulative aneurysm-related mortality of approximately 20% when including early deaths and 6% when excluding early deaths. These findings suggest that most aneurysm-related deaths occurred within the early postoperative interval, whereas late mortality was predominantly due to non-aneurysm causes.

The estimated freedom from type Ia endoleaks of 91% at 5 years is reassuring, suggesting satisfactory durability of the proximal seal in most cases. However, it should be acknowledged that approximately 30% of reinterventions in this cohort were related to endoleaks or chimneys, emphasizing that long-term durability often depends on secondary procedures. In addition, persistent late endoleaks were observed in 8% of patients, including cases of type II endoleaks with sac growth resistant to reintervention. These findings underscore the importance of regular imaging follow-up for the detection and timely management of late complications to preserve long-term clinical success. Similarly, the long-term mortality observed in this study is consistent with findings from other large multicentre registries, including the PMEG series, which also included urgent cases^8^  ^,12^. These results underline that late mortality remains substantial across all off-the-shelf and physician-modified endovascular approaches in this high-risk patient population.

Notably, no target vessel occlusions were reported within the first 30 days, demonstrating the immediate effectiveness of chimney grafts in maintaining end-organ perfusion. The late chimney occlusion rate of 6.7% is relatively low, with all occlusions affecting renal arteries and particularly the LRA, with a mean time to occlusion of 20 months. Additionally, the variability in post-procedural antiplatelet therapy, with only 42.3% of patients receiving SAPT, may have influenced these results.

The inability to successfully recanalize occluded chimneys in all attempted cases raises concerns about the long-term patency of target vessels and the potential for end-organ dysfunction. The estimated target vessel patency of 87% at 5 and 7 years is encouraging, but highlights, even more, the importance of close follow-up and monitoring of renal function in these patients, as well as the need for further optimization of chimney stent design and implantation techniques.

Although late reinterventions were required in 16.1% of patients, the majority were for type Ia and Ib endoleaks, highlighting the need for refined device design^18^ and meticulous planning^19,20^ to optimize long-term outcomes. Overall, aneurysm sac behaviour, including after reinterventions, was favourable in 45% of patients with significant shrinkage; however, sac enlargement persisted in 10% of cases despite reinterventions. The significant reduction in mean sac diameter at follow-up compared with preoperative measurements (*P* < 0.001) demonstrates the procedure’s effectiveness in achieving aneurysm control in appropriately selected patients^21^. Finally, the considerable variation in both the types of aortic stent graft and the stent grafts used for the chimneys is noteworthy.

Overall, the PEACE registry results suggest that chEVAR is a valuable option for high-risk patients requiring urgent or emergency aneurysm repair, particularly in anatomically challenging cases. However, the high rates of complications, reinterventions, and mortality necessitate careful patient selection.

The significant rates of renal impairment and chimney-related complications highlight the importance of preserving renal function during and after the procedure. Strategies such as minimizing contrast use and optimizing stent positioning may help reduce renal complications. Advances in stent graft technology, including lower-profile devices and improved chimney stents with better radial force and flexibility, could also enhance long-term outcomes. Moreover, the application of current standardized materials and procedural protocols would likely further enhance outcomes in this challenging setting.

Emerging technologies, such as customized fenestrated EVAR^22^, branched devices^23^, PMEG^12^, and *in-situ* fenestrated EVAR^13^, may offer advantages over chEVAR in certain cases, particularly for patients with longer life expectancies. However, the limited availability and production time of these devices currently restrict their use in emergency settings. Customized off-the-shelf fenestrated grafts also involve more complex procedures and are especially challenging to perform in cases with severe angulated anatomies. On the other hand, the use of branched EVAR, especially in emergency cases, carries a substantial risk of spinal cord ischaemia^24^, due to the extent of aortic coverage, which must be carefully considered. When compared with contemporary experiences using off-the-shelf branched devices, such as the multicentre series by Gallitto *et al*.^25^, outcomes in the present study appear broadly comparable in terms of technical success, mortality, and target vessel stability, despite a higher proportion of ruptured cases. These findings further support the role of chEVAR as a feasible and effective option in urgent complex aneurysm repair, particularly when rapid aneurysm exclusion is required.

These factors underscore the continued relevance of chEVAR for acute presentations, ensuring it remains a critical tool in the armamentarium for managing complex aortic pathologies in emergency situations when rapid aneurysm exclusion is required and alternative off-the-shelf devices are not readily available. This selective use of chEVAR in emergency scenarios is in accordance with the 2024 guidelines of the European Society for Vascular Surgery^1^, which recommend the use of parallel graft techniques only in emergency or bailout situations and ideally limited to fewer than two chimneys.

This study is limited by its retrospective nature, which may introduce selection and reporting biases. The multicentre design, while providing a larger sample size, may also introduce heterogeneity in patient anatomy, device selection, procedural techniques, operator experience, and case distribution across centres, as all of the patient care was done according to local practices and was not protocol driven. The absence of a standardized long-term follow-up protocol across centres may have led to variable imaging intervals and incomplete data capture. In addition, cases with >10% missing data were excluded to preserve the integrity of the reported outcomes. The long study interval (2010–2021) spans major developments in endovascular technology and techniques, which may have influenced the results. The sample size, although representing one of the largest multicentre experiences in this specific setting, could still affect the strength of the findings and the possibility of type II error cannot be excluded. Finally, the lack of a control group limits direct comparison with other treatment modalities, including open repair, *in-situ* fenestrated EVAR, PMEG, or even standard EVAR.

## Collaborators

PEACE-registry Study Collaborators: Abdulrahman Husain (Department of Vascular Surgery, University Hospitals Birmingham NHS Foundation Trust, Birmingham, UK); Francesco Squizzato (Division of Vascular and Endovascular Surgery, Department of Cardiac, Thoracic, Vascular Sciences and Public Health, Padua University School of Medicine, Padua, Italy); Simona Sica (Unit of Vascular Surgery, Fondazione Policlinico Universitario A. Gemelli IRCCS, Università Cattolica del Sacro Cuore, Rome, Italy); Yamume Tshomba (Unit of Vascular Surgery, Fondazione Policlinico Universitario A. Gemelli IRCCS, Università Cattolica del Sacro Cuore, Rome, Italy); Mauro Gargiulo (Vascular Surgery, University of Bologna, DIMEC, Bologna, Italy; Vascular Surgery Unit, IRCCS Sant'Orsola, Bologna, Italy); Konstantinos Tzimkas-Dakis (Department of Vascular Surgery, Larissa University Hospital, Faculty of Medicine, University of Thessaly, 41110 Larissa, Greece); Stefanie Burger (Department of Vascular Surgery, Maastricht University Medical Centre, Maastricht, the Netherlands); Geert Willem Schurink (Department of Vascular Surgery, Maastricht University Medical Centre, Maastricht, the Netherlands); Anders Wanhainen (Division of Vascular Surgery, Department of Surgical Sciences, Uppsala University, Uppsala, Sweden); Peter Legeza (Division of Vascular Surgery, Department of Surgical Sciences, Uppsala University, Uppsala, Sweden); Andreina Monfreda (Division of Vascular and Endovascular Surgery, Cardiovascular Department, University Hospital of Trieste, Trieste, Italy); Sandro Lepidi (Division of Vascular and Endovascular Surgery, Cardiovascular Department, University Hospital of Trieste, Trieste, Italy); Marta Ascione (Department of General Surgery and Surgical Specialties, Sapienza University of Rome, Rome, Italy); Luca di Marzo (Department of General Surgery and Surgical Specialties, Sapienza University of Rome, Rome, Italy); Fino Gianluigi (Vascular and endovascular surgery unit university of Perugia, ospedale S. Maria della Misericordia, Perugia, Italy).

## Supplementary Material

znaf278_Supplementary_Data

## Data Availability

The data that support the findings of this study are available from the corresponding author upon reasonable request.
