# Supplementary material for: Parallel Endografting And Chimney Endovascular (PEACE) registry outcomes in emergency repair of complex abdominal aortic aneurysms
Source: Br J Surg. 2025 Dec 30;113(1):znaf278. doi: 10.1093/bjs/znaf278 (PMC12750328; doi:10.1093/bjs/znaf278)
Supplement: znaf278_Supplementary_Data [file znaf278_supplementary_data.zip › Suppl.docx]

**Supplementary Table 1.** Cases performed using chimney EVAR on the acute setting of ruptured or symptomatic complex AAA in 12 academic centers.

| **Center** | **Cases, No. (%)** |
| --- | --- |
| Vascular Center, Skåne University hospital, Malmö, Sweden | 48 (40) |
| Department of Vascular Surgery, University Hospitals Birmingham NHS, UK | 22 (18) |
| Department of Cardiac, Thoracic, Vascular Sciences and Public Health,  Padua University School of Medicine, Italy | 8 (7) |
| Unit of Vascular Surgery, Fondazione Policlinico Universitario A. Gemelli IRCCS, Università Cattolica del Sacro Cuore, Rome, Italy | 7 (6) |
| IRCCS Azienda Ospedaliero-Universitaria di Bologna, Italy | 6 (5) |
| Department of Vascular Surgery, Larissa University Hospital, Greece | 6 (5) |
| Department of Vascular Surgery, Maastricht University Medical Centre, Maastricht, the Netherlands. | 6 (5) |
| Division of Vascular Surgery, Department of Surgical Sciences, Uppsala University, Uppsala, Sweden. | 6 (5) |
| Division of Vascular and Endovascular Surgery, Cardiovascular Department, University Hospital of Trieste, Trieste, Italy. | 4 (3) |
| Department of General Surgery and Surgical Specialties, Sapienza University of Rome, Rome, Italy. | 3 (3) |
| Vascular and endovascular surgery unit university of Perugia, ospedale S.Maria della Misericordia, Perugia, Italy. | 1 (1) |
| German Aortic Center Hamburg, Department of Vascular Medicine, University Heart and Vascular Center UKE Hamburg | 1 (1) |
| Total | 118 (100) |

**Supplementary Table 2:** Anatomic characteristics and indications for repair of 118 patients treated with chimney EVAR on acute setting of complex AAA. Categorical variables are presented as number (%).

| **Arteries** | **Self-expandable stentgraft** | **Balloon-expandable stentgraft** | **Balloon-expandable non covered stent** | **Total (N=181)** |
| --- | --- | --- | --- | --- |
| Celiac trunk | 3 (50%) | 3 (50%) | - | 6 (3) |
| Superior mesenteric artery | 8 (22) | 29 (78) | - | 37 (20) |
| Right renal artery | 11 (17) | 54 (82) | 1 (2) | 66 (36) |
| Left renal artery | 15 (21) | 55 (76) | 2 (3) | 72 (40) |

**Supplementary Table 3:** Univariable analysis identifying independent predictors of 30-day mortality and type Ia endoleak on completion angiography in 118 patients treated with chimney EVAR for acute complex AAA.

| Parameter,  Univariate | 30-days mortality  HR | | 95% CI  Lower Upper | | P value | Type Ia EL, completion  HR | 95% CI  Lower Upper | | P value |
| --- | --- | --- | --- | --- | --- | --- | --- | --- | --- |
| Age, years | | .997 | .911 | 1.091 | .948 | 1.003 | .900 | 1.118 | .955 |
| Male sex | | .756 | .184 | 3.101 | .698 | .306 | 067 | 1.398 | .127 |
| Diameter AAA, mm | | 1.022 | .990 | 1.056 | .175 | 1.000 | .961 | 1.039 | .983 |
| Main aortic device oversizing, % | | 1.045 | .959 | 1.138 | .316 | 1.019 | .915 | 1.135 | .726 |
| Use of occlusion balloon | | 3.457 | .914 | 13.079 | .068 | .773 | .157 | 3.802 | .752 |
| Use of Palmaz stent | | .327 | .018 | 5.827 | .447 | 3.039 | .208 | 44.404 | .417 |
| Intentional coverage of visceral vessel | | 1.098 | .306 | 3.938 | .886 | 2.757 | .473 | 16.077 | .260 |
| Number of chimneys, 1 | | .895 | .282 | 2.837 | .895 | 1.321 | .226 | 7.719 | .758 |
| Number of chimneys, 2 | | .000 | .000 | .000 | .999 | 29.612 | 1.108 | 791.35 | **.043** |
| Number of chimneys, 3 | | .808 | .000 | .000 | 1.00 | 1.460 | .806 | 2.643 | .212 |
| EVAR device, Gore Flex | | .000 | .000 | .000 | .998 | 2.039 | .118 | 35.279 | .624 |
| EVAR device, Gore Excluder | | .238 | .061 | .930 | **.039** | .600 | .050 | 7.193 | .687 |

**Supplementary Table 4:** Summary of all early reinterventions performed within 30 days postoperatively. Categorical variables are presented as number (%).

| **Early reinterventions** | **N=23 (%)** |
| --- | --- |
| Chimney related | 7 (7) |
| SMA relining and stenting | 3 (3) |
| embolization of type 1a EL due to gutters | 2 (2) |
| CT relining + re-PTA RRA chimney | 1 (1) |
| LRA relining and extension | 1 (1) |
| Aortic related | 2 (2) |
| rupture of aortic neck – Palmaz stent | 1 (1) |
| aortic sac coiling | 1 (1) |
| Non-aortic/non-chimney related | 14 (12) |
| suture of access site pseudoaneurysm | 4 (3) |
| bowel resection | 3 (3) |
| abdominal VAC change - repetitively | 2 (2) |
| thrombectomy + TEA of access groin | 2 (2) |
| explorative laparotomy – no findings | 1 (1) |
| splenectomy due to intraabdominal bleeding | 1 (1) |
| celiac trunk ligation due to perforation | 1 (1) |

**Supplementary Table 5:** Summary of all later reinterventions performed postoperatively. Categorical variables are presented as number (%).

| **Late reinterventions** | **(N=25)** |
| --- | --- |
| Chimney related | 7 (28) |
| Unsuccessful try for revascularization of occluded RRA chimney | 3 |
| Coil embolization of gutters due to TIaELs | 2 |
| SMA relining and stenting | 1 |
| SMA stenting due to compression from renal chimney | 1 |
| Aortic related | 16 (64) |
| Proximal extension due to TIaELs | 4 |
| Type II EL embolization | 3 |
| Distal extension due to TIbELs | 6 |
| Endoanchors in proximal sealing zone due to TIaEL# | 1 |
| Open conversion due to TIaEL post endoanchors# | 1 |
| Open conversion due to graft infection | 1 |
| Non-aortic/non-chimney related | 2 (8) |
| Abdominal hernia and net | 1 |
| Embolization of iatrogenic bleeding during other reintervention | 1 |

#: refers to same patient, *: refers to same patient

**Supplementary Figure 1: Estimated freedom from type Ia endoleak (TIaELs) for patients who underwent urgent chimney-EVAR for a complex abdominal aortic aneurysm (AAA).**

**
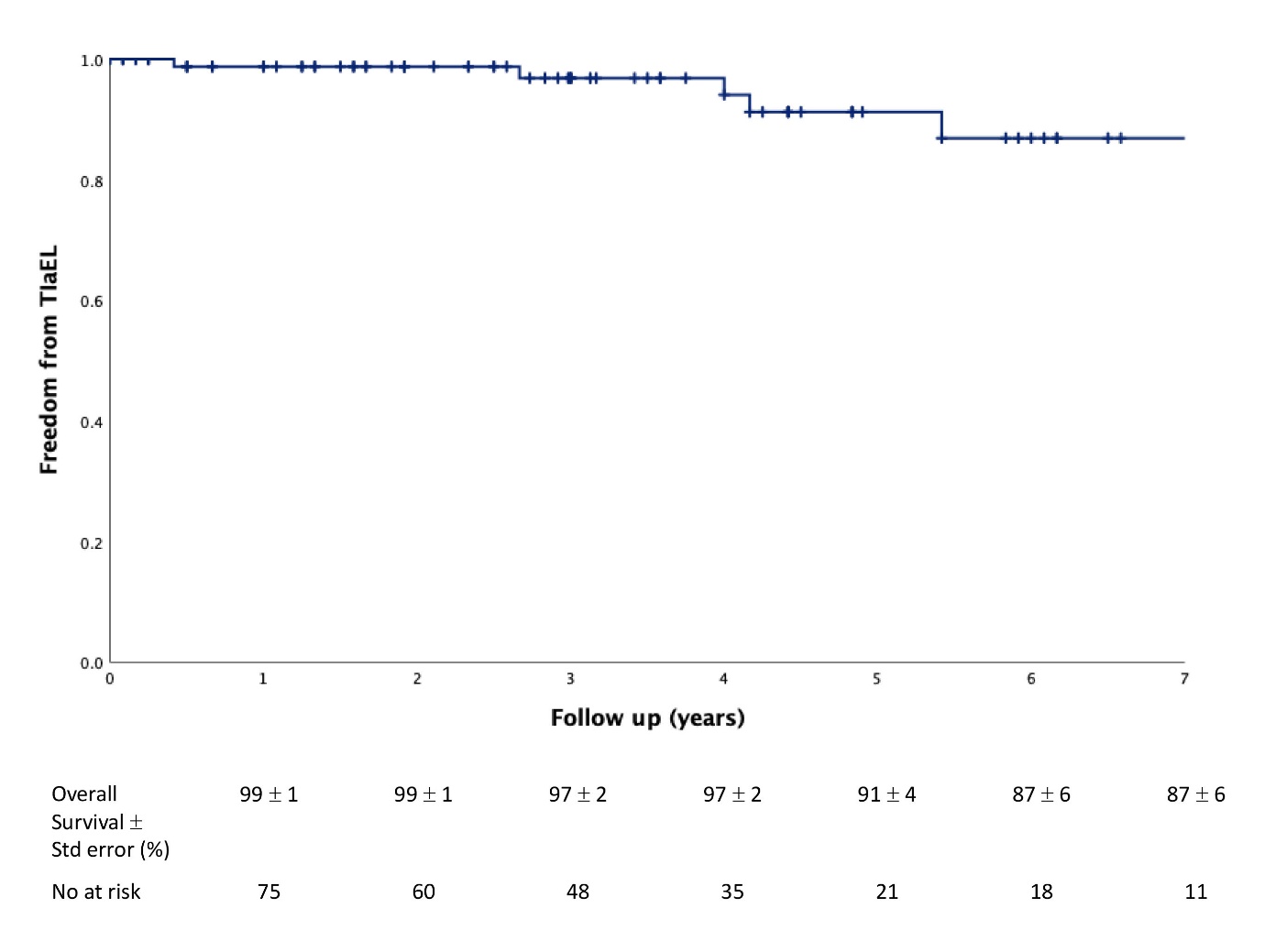
**

**Supplementary Figure 2: Estimated target vessel patency for patients who underwent urgent chimney-EVAR for a complex abdominal aortic aneurysm (AAA).**

**

**

**Supplementary Figure 3: Estimated primary, blue line, and secondary, red line, clinical success for patients who underwent urgent chimney-EVAR for a complex abdominal aortic aneurysm (AAA).**

**
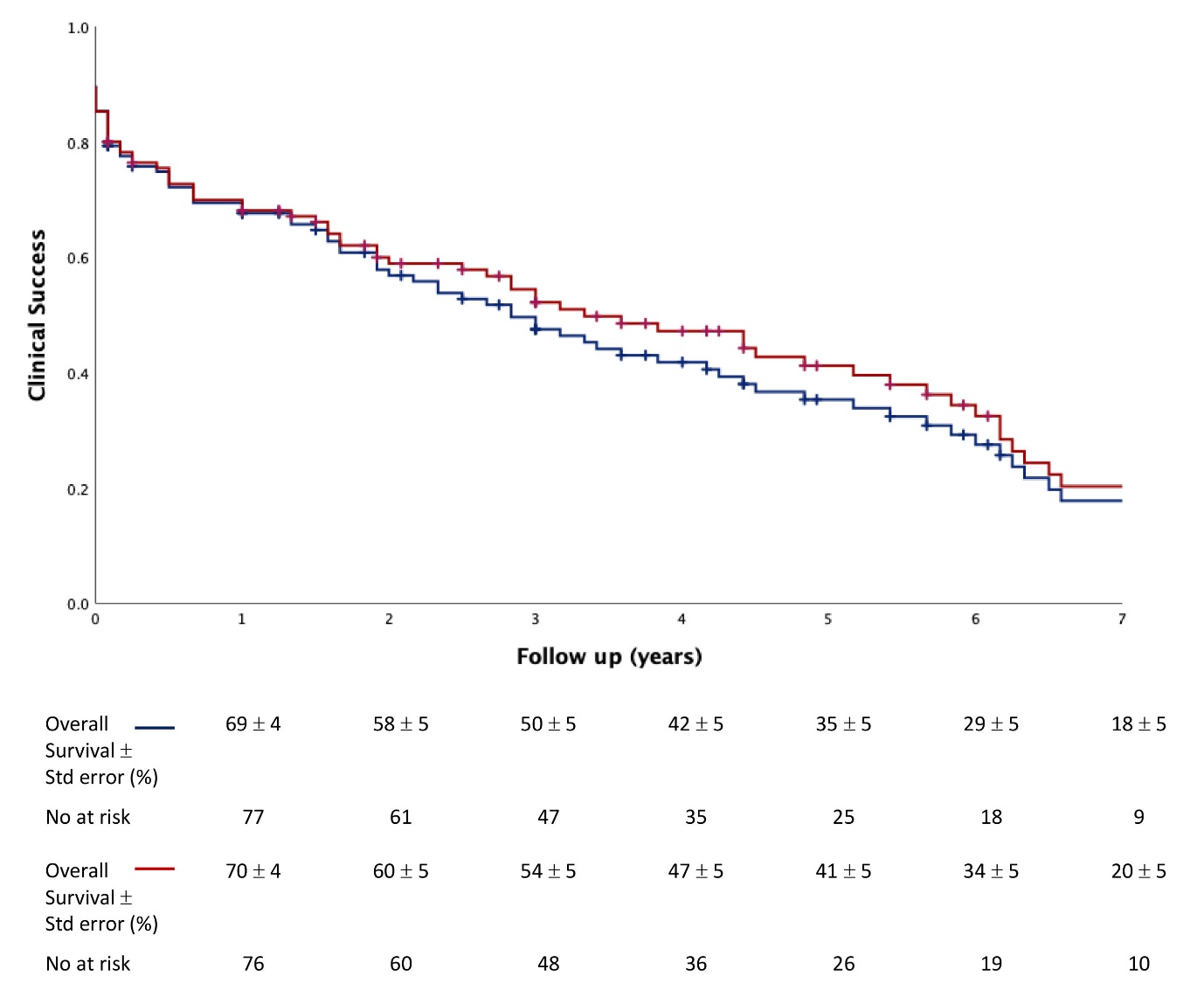
**
