# Supplementary figures and images for: Parallel Endografting And Chimney Endovascular (PEACE) registry outcomes in emergency repair of complex abdominal aortic aneurysms
Source: Br J Surg. 2025 Dec 30;113(1):znaf278. doi: 10.1093/bjs/znaf278 (PMC12750328; doi:10.1093/bjs/znaf278)

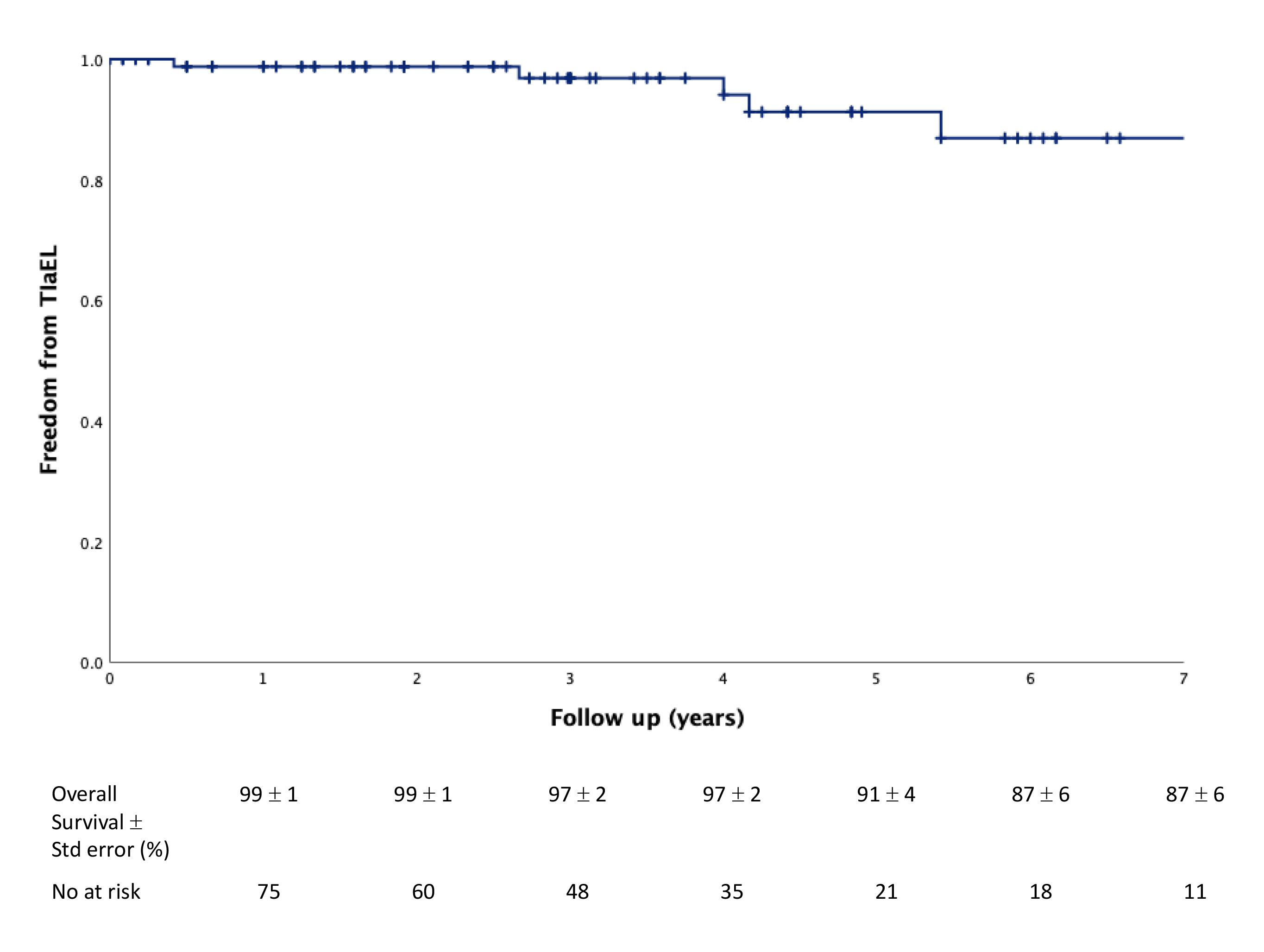

Supplement: znaf278_Supplementary_Data [file znaf278_supplementary_data.zip › Supplementary Figure 1.jpg]

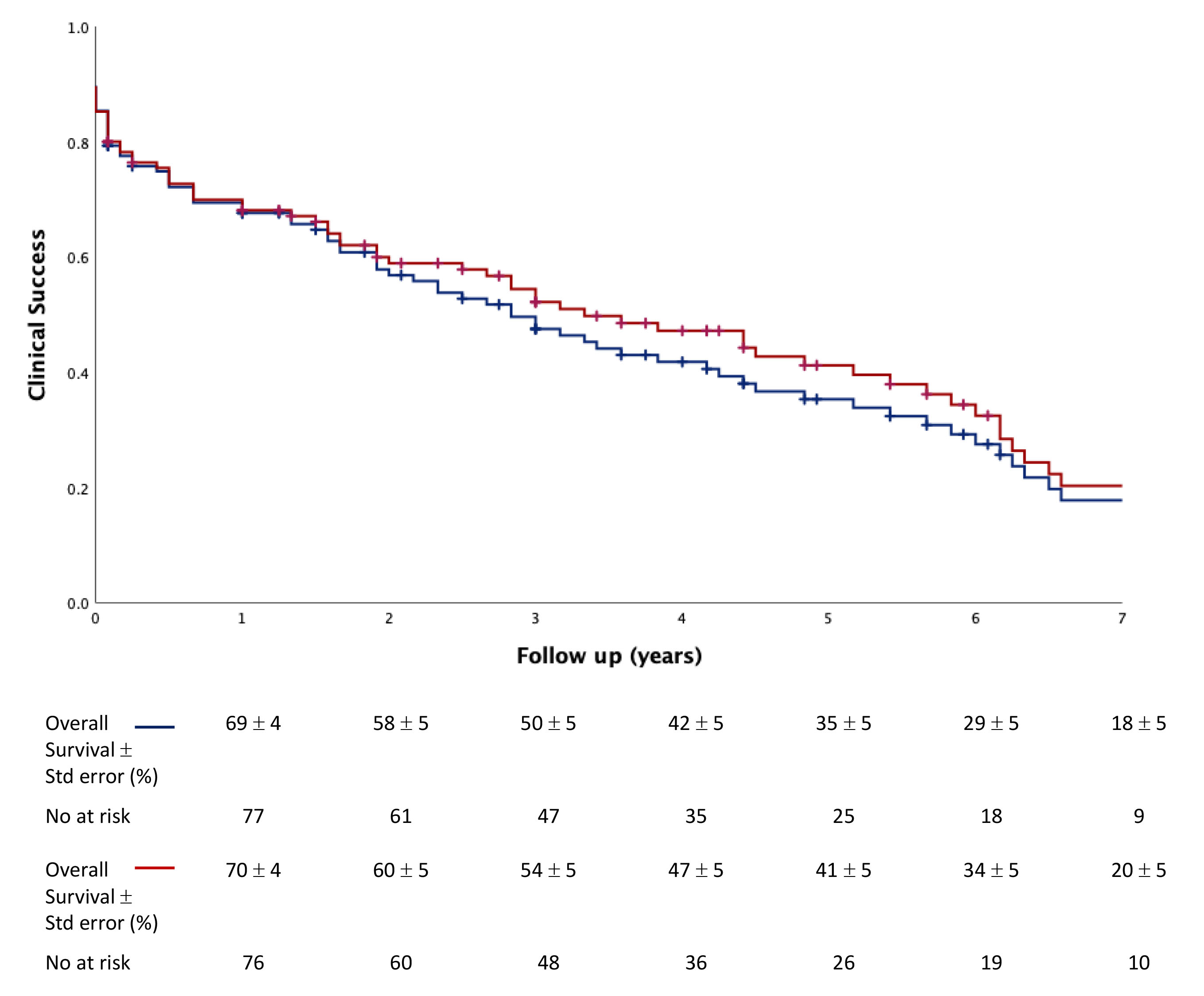

Supplement: znaf278_Supplementary_Data [file znaf278_supplementary_data.zip › Supplementary Figure 3.jpg]
